# No-tillage with straw mulching promotes the utilization of soil nitrogen by rice under wheat–rice and oilseed rape–rice cropping systems

**DOI:** 10.3389/fpls.2023.1170739

**Published:** 2023-05-08

**Authors:** Fengjun Yan, Wei Zhou, Yongjian Sun, Changchun Guo, Kaihong Xiang, Na Li, Zhiyuan Yang, Yunxia Wu, Qiao Zhang, Yuanyuan Sun, Xiyao Wang, Jun Ma

**Affiliations:** ^1^ Rice Research Institute of Sichuan Agricultural University, Wenjiang, Chengdu, China; ^2^ Crop Ecophysiology and Cultivation Key Laboratory of Sichuan Province, Wenjiang, China; ^3^ Institute of Plateau Meteorology, China Meteorological Administration, Chengdu, China; ^4^ The Rural Revitalization Research Institute of Sichuan Tianfu New Area, Chengdu, China

**Keywords:** straw incorporation, conservation tillage, paddy-upland rotation, isotopic labeling, nitrogen uptake

## Abstract

**Introduction:**

To investigate the effects of no-tillage with straw mulching on the absorption and utilization of soil nitrogen (N), fertilizer N, and straw N by rice under paddy-upland rotations.

**Methods:**

A field experiment with three cropping systems: fallow–rice rotation without straw mulching (FRN), wheat–rice rotation with wheat mulching in rice season (WRS), and oilseed rape–rice rotation with oilseed rape straw mulching in rice season (ORS) was conducted from 2015 to 2017, along with a mini-plot experiment with ^15^N-labeled urea and straws, which was conducted in 2017.

**Results:**

No-tillage with straw reduced rice N uptake up to 20 days after transplanting, the total amount of fertilizer N uptake of WRS and ORS rice plants was 46.33 and 61.67 kg/ha, respectively, which was 9.02 and 45.10% higher than that of FRN plants. Soil N was the main source for rice growth, followed by fertilizer N. Soil N uptake by WRS and ORS rice plants was 21.75 and 26.82% higher than that of FRN plants, accounting for 72.37 and 65.47%, respectively, of the total N accumulated in rice plants. Straw mulching increased the N utilization efficiency of tillering, panicle, and total fertilizer by 2.84–25.30%; however, base fertilizer was dependent on straw mulching. The total amount of N released from WRS and ORS straw mulching in the rice season was 34.97 and 24.82 kg/ha, respectively; however, only 3.04 and 4.82% of it was absorbed by the rice plants, accounting for only 0.62 and 0.66% of the total accumulated N.

**Discussion:**

No-tillage with straw mulching under paddy-upland rotations increased the N utilization of rice, especially for the absorption of soil N. These results provide theoretical information for the effective utilization of straw and rational N application practices in rice-based cropping systems.

## 1 Introduction

Rice is an important food crop and approximately 50% of the world’s population depends on rice as a staple food. Alternating the rice crop with upland crops, known as paddy-upland rotation, is the most efficient cropping system for ensuring global food security, especially in Asia (Zheng et al., 2016). However, unreasonably intense cropping cultivation leads to a decrease in soil fertility and crop yield and an increase in the usage of chemical fertilizers (Srinivasan et al., 2012; Nishida, 2016; Nandan et al., 2019). For example, rice-wheat cropping systems, the most popular paddy-upland rotations, have shown a clear slowing or stagnation in crop yield, which is related to the cycling of soil N (Ram et al., 2016; Zhou et al., 2021). Moreover, the amount of N fertilizer (as pure N) applied worldwide in 2012 was nearly 186 times higher than that in 1961. China’s N input generally exceeds 180 kg/ha for rice season; however, the N utilization rate in rice is only approximately 30% (Tian et al., 2001; Pan et al., 2017). Many inorganic fertilizers are lost into the atmosphere, surface water, and groundwater, leading to the pollution of the air and water environments and the wastage of nonrenewable resources (Wang et al., 2014). Therefore, stabilizing the rice yield with less N input or increasing the rice yield without increasing the N input is the focus of agricultural research.

No-tillage is a variant of conservation tillage that is generally used to improve soil properties and crop yield (Shakoor et al., 2020; Thapa et al., 2023). However, sustainable agricultural production cannot be achieved through an isolated practice of no-tillage; it must be combined with crop residue retention (Rafael et al., 2021). Crop straw is rich in N and other nutritional elements, but traditional straw disposition (directly burned or arbitrarily stacked) causes serious environmental pollution and leads to higher wastage of resources. By contrast, no-tillage with straw mulching increases soil quality and crop yield by regulating N cycling in the soil and N uptake by crops (Yang et al., 2020; Yang et al., 2022a; Yang et al., 2022b). However, straw mulching may lead to higher N consumption, increase its immobilization, and accumulate allelochemicals in the prior decomposition period, which causes N stress and inhibits root growth and N absorption during the initial growth period of crops (Liu et al., 2007; Yan et al., 2019; Singh et al., 2022). Over a larger 12-year rotation trial, Flower et al. (2022) revealed that whether straw mulching has a positive or negative impact on crop growth depends on the crop and straw types, and environmental conditions such as soil moisture. Therefore, we hypothesized that different crop types and the special water management alternating between wet and dry conditions will change soil properties, resulting in different responses to conservation tillage practices in paddy-upland rotations.

Previous studies on conservation tillage have mainly focused on dryland crops; however, studies on whether no-tillage in combination with straw mulching can promote N uptake by rice plants under paddy-upland rotations with less N input are scarce. Therefore, the present study set up a 3-year field experiment with three cropping systems under no-tillage in combination with low chemical N application. To determine the source of N absorbed by the rice, a mini-plot experiment using ^15^N-labeled urea and straw was performed in 2017. The aim of this study was to elucidate the principles of N release from wheat and oilseed rape straw and its effects on N uptake by rice plants under paddy-upland rotation with no-tillage, which could provide theoretical support for the sustainable production of rice-based cropping systems with N reduction.

## 2 Materials and methods

### 2.1 Experimental site

**Figure 1:**
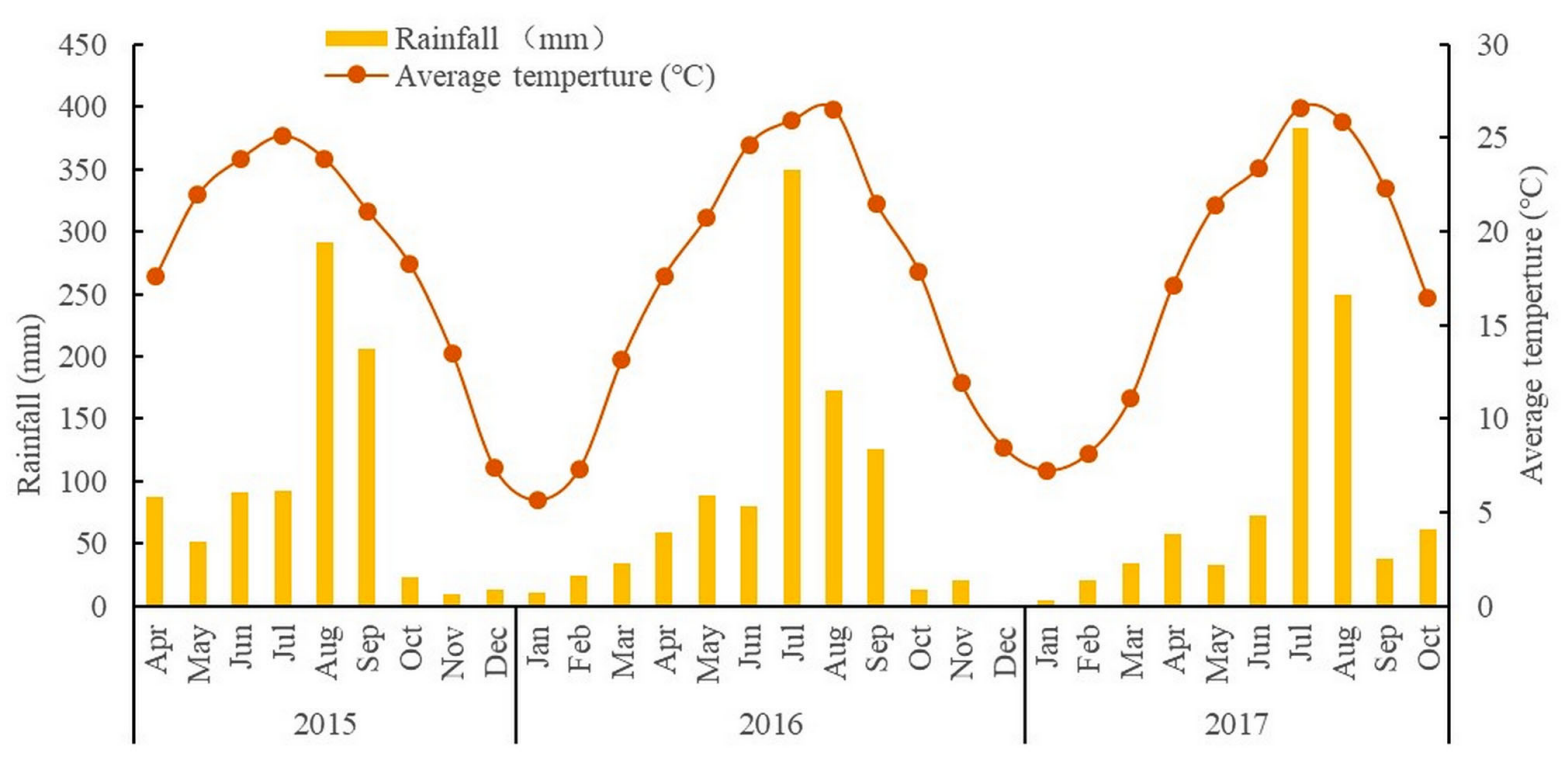
The average temperature (°C) and rainfall (mm) during 2015-2017.

The experiment was performed at a farm of the Rice Research Institute of Sichuan Agricultural University Chengdu, China (30°35′N, 103°45′E), during 2015–2017. The soil had a sandy loam texture with a total N of 1.96 g/kg, organic matter of 26.00 g/kg, available N of 29.13 mg/kg, available P of 81.60 mg/kg, and available K of 85.98 mg/kg at the time of experiment initiation. The region is classified as humid subtropical with a monsoon climate, and the meteorological data for the experimental years, which were measured at a weather station near the experimental site, are shown in **Figure 1**.

### 2.2 Experimental design and management

The experiment comprised three treatments in a single-factor randomized block design with three replicates during 2015–2017. The treatments were designed as follows: wheat–rice rotation with wheat straw mulching during the rice season (WRS), oilseed rape–rice rotation with oilseed rape straw mulching in the rice season (ORS), and fallow–rice rotation without straw mulching (FRN), which served as the control (**Table 1**). The plot size was 4.8 m × 3.3 m, and plots were separated by a 0.4 m wide alley with a plastic film inserted into the soil to form a barrier. Wide–narrow row spacing cultivation was used for rice plantation in the present study. Wheat and oilseed rape straws were cut into 5–10 cm long pieces and mulched on a wide row immediately after rice transplantation. Alternate dry/wet irrigation was applied, and the seedlings were transplanted in shallow water (1–2 cm). The field was submerged in a 2 cm water layer for 5–7 days after transplanting to ensure that the seedlings turned green and survived. Thereafter, the water was drained from the field until booting, and the soil water content accounted for 70–80% of the saturated water content. The field was dried during the ineffective tillering stage. The field was again submerged in a 1–3 cm water layer at the booting stage. Irrigation with 3 mm water was carried out in the bolting stage of wheat and oilseed rape, and rain-fed irrigation was applied in other growth periods. The total N applied in the rice and upland crop seasons was 135 and 48 kg/ha, respectively, which was much lower than the 180 and 120 kg/ha applied in conventional cultivation reported by Peng et al. (2002). Details of other cultivation measures and fertilizer applications are shown in **Tables 1**, **2**.

**Table 1:** Field management for test crops.

Crop	Cultivar	Tillage	Planting method	Spacing (cm×cm)	Plants per hill	Crop season
Wheat	Sumai 375	No tillage	Hill-direct-seeding	20×10;	3-5	October- June
Oilseed rape	Chuanyou 58	No tillage	Hill-derect-seeding	30×20	3-5	October-June
Rice	Yixiang-3724	No tillage	Artificial transplanting	(40 + 26.5)×16.7	1	June-September

**Table 2:** Detailed application of fertilizers for test crops in field experiment (kg/ha).

Crop	Application amount			Base fertilizer				First top dressing		Second top dressing	
	N	P_2_O_5_	K_2_O	CF	Urea	SSP	KCl	CF	Urea	CF	Urea
Wheat	48	48	48	64	–	–	–	128	–	128	–
Oilseed rape	48	48	48	64	–	–	–	128	–	128	–
Rice	135	67.5	135	–	88	562.5	225	–	88	–	117.4

CF, compound fertilizer (the content of N, P_2_O_5_, and K_2_O all was 15%); SSP, calcium superphosphate; KCl, muriate of potash. Base fertilizer was applied before plant transplantation or sowing; first top dressing was applied at the early tillering stage for rice, at the jointing stage for wheat, and at the wintering stage for oilseed rape; second fertilizer top dressing was applied at the panicle initiation stage for rice, at the bolting stage for wheat and at oilseed rape.

The ^15^N mini-plot experiment was performed along with a field experiment in 2017. Four metal frames without bottoms (80 cm long × 70 cm wide × 50 cm high) were installed 30 cm deep in the soil and 20 cm above the soil surface around 10 adjacent rice plants in each field plot. The ^15^N-labeled urea and straw were applied as described in **Table 3**. The ^15^N abundance and total N content of labeled wheat and oilseed rape straw were 0.763 atom% and 0.749% and 0.634 atom% and 0.620%, respectively. The application times of fertilizers and other management practices were the same as those in the field experiment.

**Table 3:** The usage of ^15^N-labeled urea and straw in the mini-plots.

Mini-plot	Base N	Tillering N	Panicle N	Straws
1^st^	**^15^N**	^14^N	^14^N	^14^N
2^nd^	^14^N	**^15^N**	^14^N	^14^N
3^rd^	^14^N	^14^N	**^15^N**	^14^N
4^th^	^14^N	^14^N	^14^N	**^15^N**

### 2.3 Indices and measurement methods

#### 2.3.1 N release from straws

After rice transplanting, 4–6 bags of 0.4 mm mesh nylon filled with 30–40 g of straw was randomly mulched in each plot of WRS and ORS. The straw bags were collected on days 20 and 30 after the transplanting, heading, and mature stages (20 and 20 DAT, HS, and MS), and gently washed to remove the soil. All samples were oven-dried at 105 °C for 1 h, then at 70 °C until they reached a constant weight. Thereafter, the samples were crushed and sieved (mesh size = 0.178 mm). Total N content was determined using a FOSS-KJ8400 apparatus (FOSS, Sweden). The ^15^N abundance values were determined using mass spectrometry at the Shanghai Research Institute of Chemical Industry (Arulmozhiselvan and Beeman, 2017).

#### 2.3.2 N accumulation in plants

Three representative rice plants containing the average number of tillers in each plot were collected on day 30 DAT, HS, and MS of rice and at the MS of wheat and oilseed rape. The plants were separated into leaves, stems, sheaths, and panicles (at heading and maturity). The follow-up processing as described in section 2.3.1 was performed to determine the total N content and ^15^N abundance values. The amount of ^15^N originating from the labeled urea and straw was determined as described by Beeman and Arulmozhiselvan (Du et al., 2009).

### 2.4 Data analysis

The data were statistically analyzed to test the level of significance using a single-factor randomized block design. Analysis of variance was performed using SPSS Version 12.0 and Sigma Plot 12.0 to test the effects of treatments and interactions.

## 3 Results

### 3.1 N uptake by rice plants

**Figure 2:**
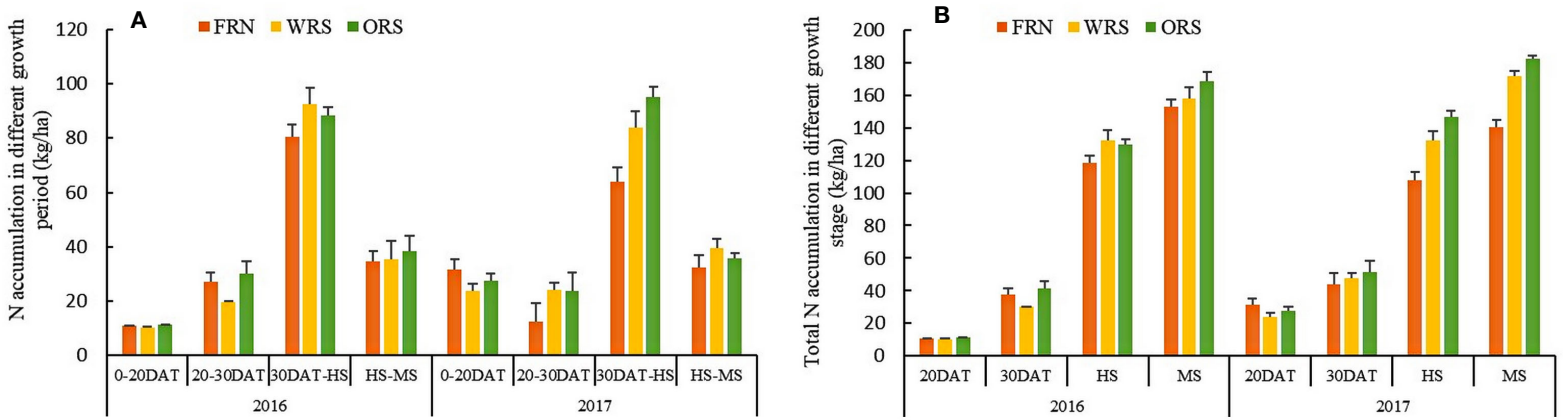
Rice nitrogen accumulation in rice growth **(A)** periods and total nitrogen accumulation in differenct growth stage **(B)**. FRN, fallow-rice rotation with no straw mulching; WRS, wheat-rice rotation with wheat straw mulching; ORS, oilseed rape-rice with oilseed rape straw mulching; DAT, days after rice transplanting; HS, heading stage; MS, mature stage.

N accumulation increased with the growth and development of rice plants, but the amount of N uptake at 30 DAT-HS was the highest, followed by HS-MS (**Figure 2**). Straw mulching promoted the absorption of N by rice plants, especially after 30 days of transplanting, and the effect of ORS was stronger than that of WRS. In 2016, the rice N uptake of WRS at 20–30 DAT and 30 DAT-HS was significantly decreased by 27.56% and increased by 14.91%, respectively, compared to that of FRN, whereas the rice N uptake of ORS was 3.35–11.64% higher than that of FRN during the entire rice growth period. The rice N accumulation of ORS was 4.21% lower at 30 DAT-HS but 8.49, 54.12, and 9.32% higher at 0–20 DAT, 20–30 DAT, and HS-RS, respectively, compared to that of WRS. In 2017, except for 0–20 DAT, rice N uptake of WRS and ORS was 10.10–95.22% higher than that of FRN. Rice N uptake of ORS was 0.92 and 9.70% lower at 20–30 DAT and HS-RS, respectively, but 14.86 and 13.12% higher at 0–20 DAT and 30 DAT-HS, respectively, compared to that of WRS. Although the amount of N accumulation differed among the three treatments at different stages, the total amount of N accumulated at the mature stage was the highest for ORS, followed by that for WRS, which was 10.09–29.67% and 2.99–22.19% higher than that of FRN.

### 3.2 N sources of rice plants

**Figure 3:**
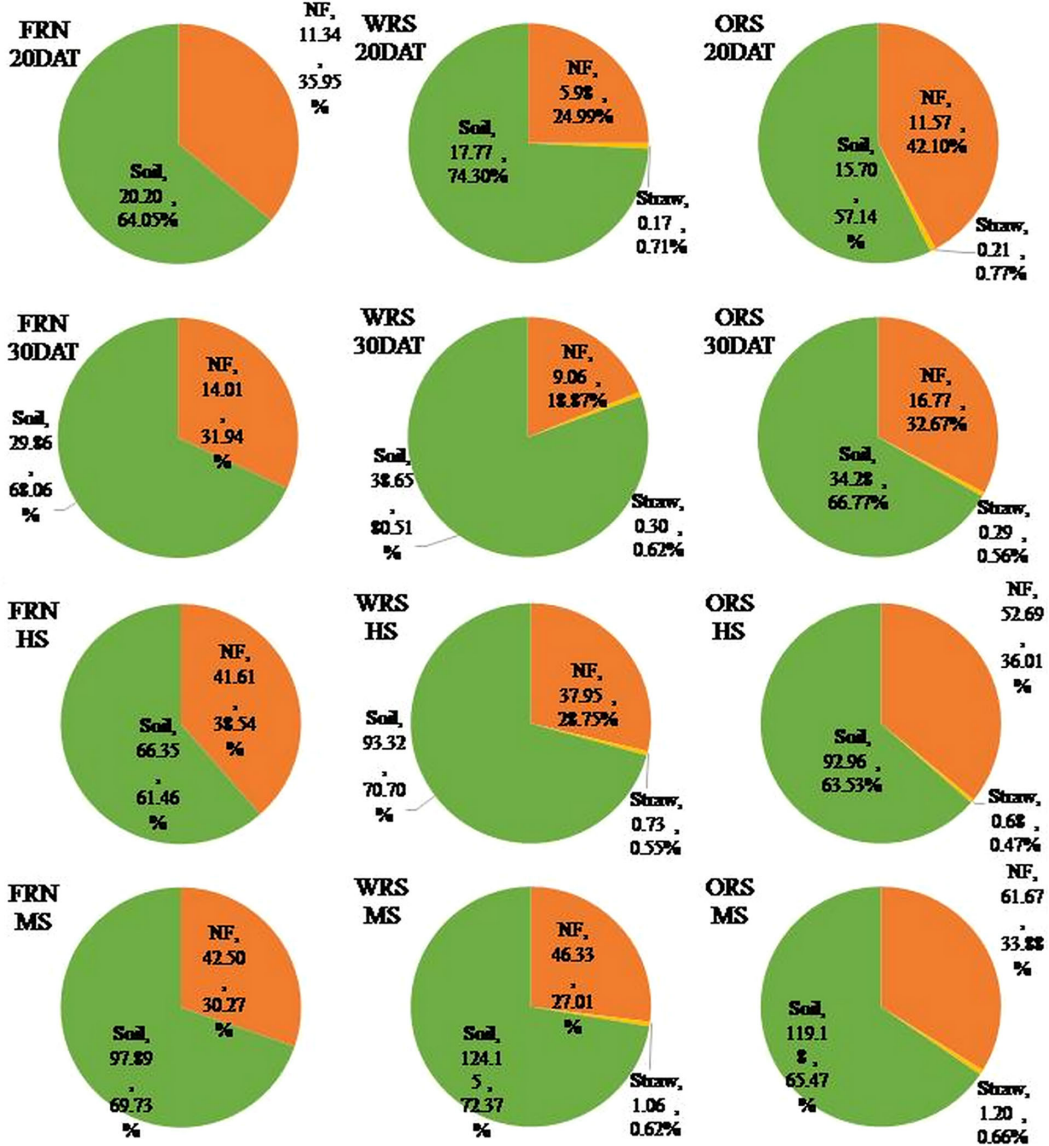
The source and its proportion of nitrogen in rice plants. FRN, fallow-rice rotation with no straw mulching; WRS, wheat-rice rotation with wheat straw mulching; ORS, oilseed rape-rice with oilseed rape straw mulching; DAT, days after rice transplanting; HS, heading stage; MS, mature stage; NF, fertilizer nitrogen.

Total N accumulation in rice plants increased in WRS mainly because of the increase in the uptake of soil N, whereas in ORS it was because of the synchronous increase in the uptake of soil N and fertilizer N (**Figure 3**). Straw mulching significantly promoted the uptake of N from the soil by rice plants from 30 DAT to MS. The amount of soil N uptake by rice plants with straw mulching was 11.68–12.01% lower than that of FRN at 20 DAT, but 14.79–29.43%, 40.11–40.64%, and 21.75–26.82% higher than that of FRN at 30 DAT, HS, and MS, respectively. The effects of oilseed rape and wheat straw mulching on N fertilizer uptake by rice plants were significantly different. The amount of fertilizer N uptake by WRS rice plants from 20 DAT to HS was lower than that of FRN, whereas ORS promoted fertilizer uptake by rice plants throughout the growth stages. As a result, the total amount of fertilizer N uptake by rice plants of WRS and ORS was 46.33 and 61.67 kg/ha, which was 9.02 and 45.10% higher than that of FRN, respectively. Although straw mulching promoted N absorption by rice plants, the amount of straw N absorbed by rice plants was 1.06–1.20 kg/ha, only accounting for 0.62–0.66% of the total N uptake.

### 3.3 Release and utilization of straw N

**Figure 4:**
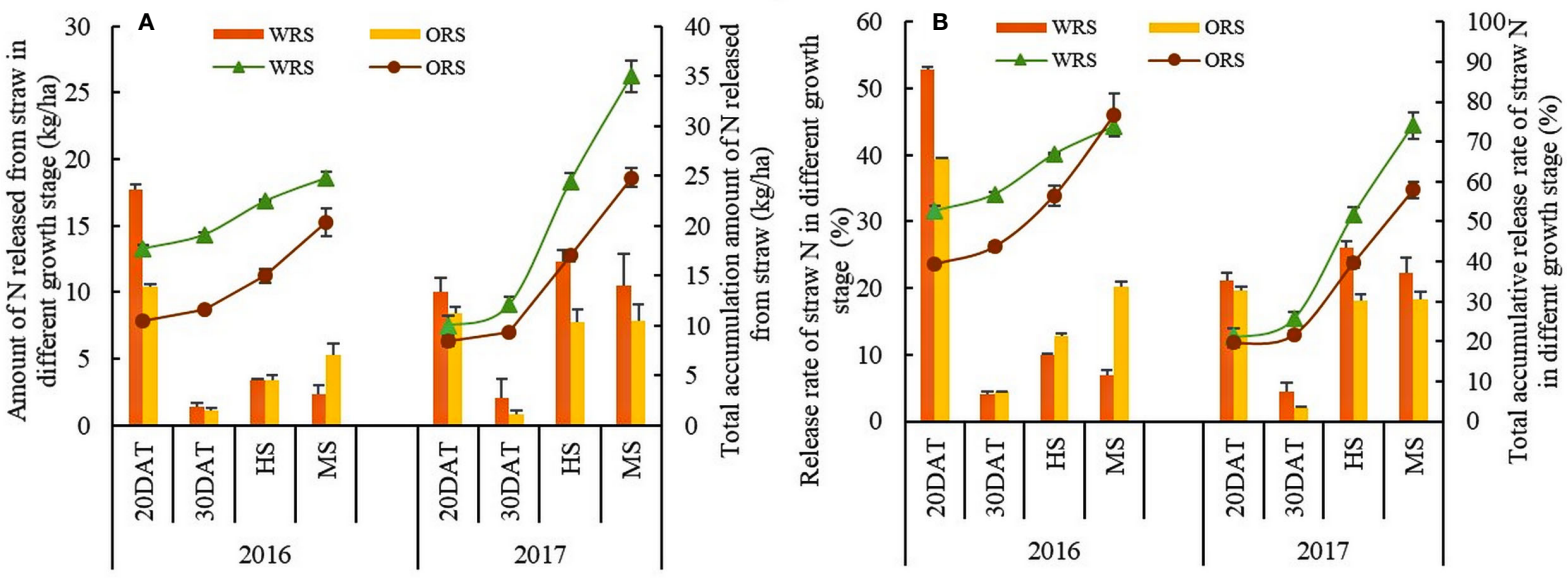
Straw nitrogen amount and accumulation amount of N release **(A)**, and straw release and accumulation release rate of straw **(B)** in rice growth stages. WRS, wheat-rice rotation with wheat straw mulching; ORS, oilseed rape-rice with oilseed rape straw mulching; DAT, days after rice transplanting; HS, heading stage; MS, mature stage.

**Figure 5:**
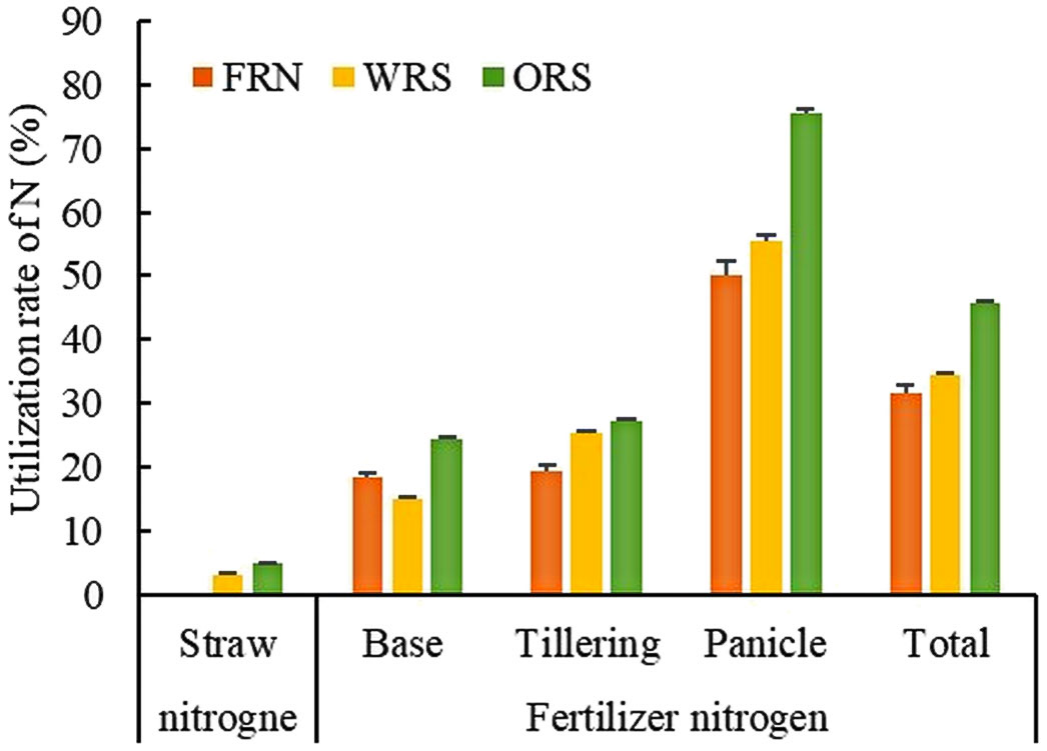
Utilization rate of nitrogen from straw and fertilizers. FRN, fallow-rice rotation with no straw mulching; WRS, wheat-rice rotation with wheat straw mulching; ORS, oilseed rape-rice with oilseed rape straw mulching.

N release from straw increased gradually with the growth of rice plants (**Figure 4**). Except for the release rate at MS in 2016, the cumulative release amount and rate of WRS were higher than those of ORS at all stages. The total amount of N released from WRS was 24.76 and 34.97 kg/ha, which was 10.1 and 40.87% higher than that from ORS in 2016 and 2017, respectively. The N release amount and rate of WRS and ORS first decreased and then increased, and the release rate was the highest at 20 DAT, accounting for 21.20–52.77% and 19.68–39.41% of the total release in WRS and ORS, respectively. For different stages, the amount of straw N released from WRS at 0–20 DAT and 20–30 DAT was 69.70 and 19.54% higher than that from ORS in 2016, respectively, which significantly increased by 34.62–149.78% during the entire rice growth period in 2017. Only 3.04–4.82% was absorbed and utilized by rice plants out of the total 20.31–34.97 kg/ha of N released by straw mulching, and most of it remained in the soil (**Figure 5**). The amount of N released by WRS was higher than that released by ORS; however, the utilization rate was 1.78% lower than that of ORS.

### 3.4 Utilization of fertilizer N applied at different stages

**Figure 6:**
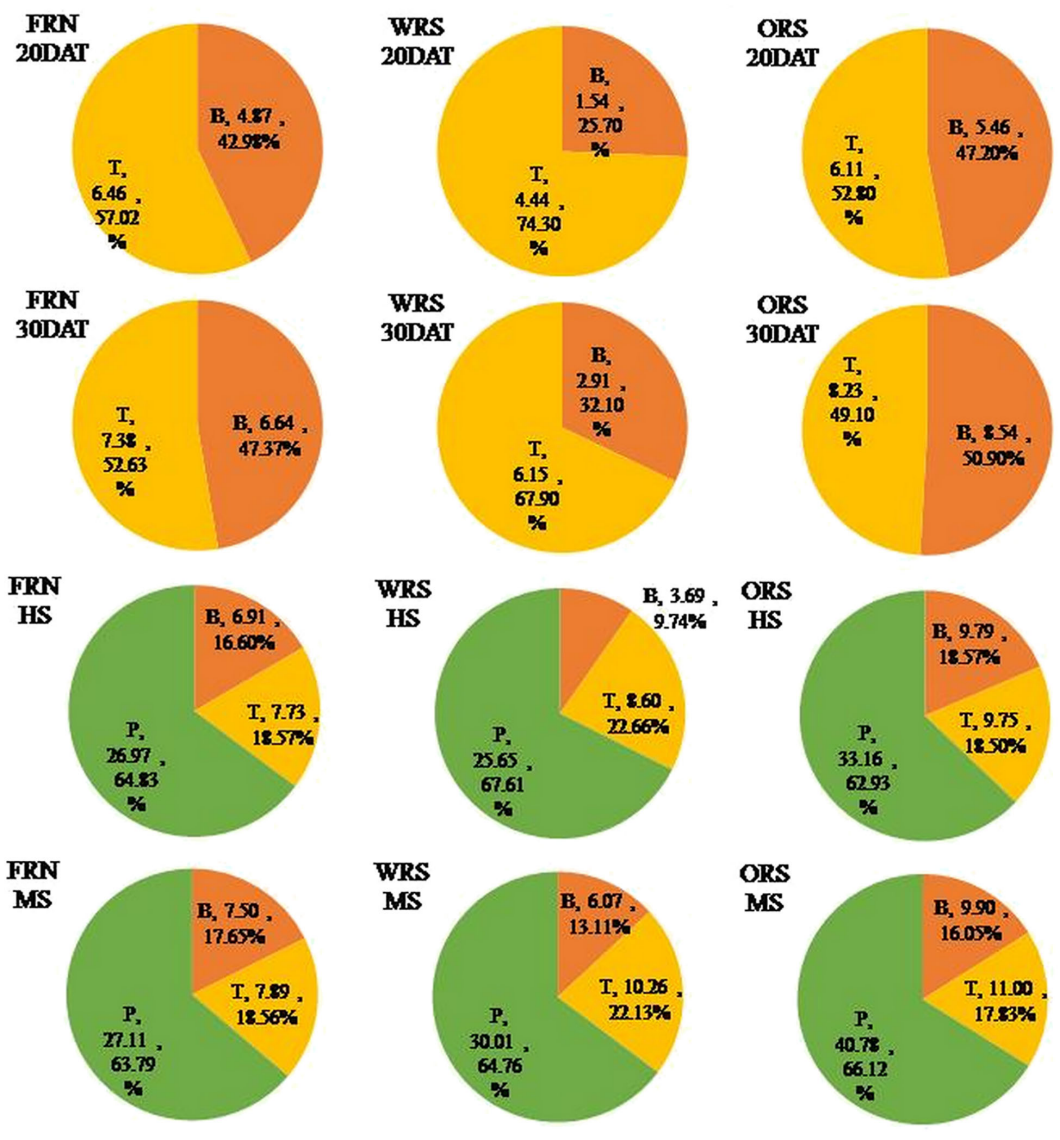
The amount and proportion of different N fertilizer uptake by rice plants. FRN, fallow-rice rotation with no straw mulching; WRS, wheat-rice rotation with wheat straw mulching; ORS, oilseed rape-rice with oilseed rape straw mulching; DAT, days after rice transplanting; HS, heading stage; MS, mature stage; B, base nitrogen fertilizer; T, tillering nitrogen fertilizer; P, panicle nitrogen fertilizer.

N use efficiency increased with straw mulching; however, the absorption and utilization rates of N applied to rice at different growth stages were significantly different (**Figure 6**). N uptake of base fertilizer in WRS decreased by 19.03–68.48% at different growth stages, whereas that of tillering and panicle fertilizer in WRS first decreased and then increased, reaching 30.03 and 10.66% at MS, respectively, compared to that of FRN. N uptake of base, tillering, and panicle fertilizer in ORS increased at different growth stages (except for tillering fertilizer at 20 DAT), reaching 12.01–41.69%, 5.53–39.44%, and 22.94–50.39%, respectively, compared to that in FRN. Therefore, the N utilization rate of the base fertilizer of WRS and ORS decreased by 3.52% and increased by 5.91%, respectively, whereas that of tillering and panicles in WRS and ORS increased by 5.85 and 5.35%, and 7.68 and 25.30%, respectively, compared to that in FRN, which led to the increase in total N fertilizer utilization by 2.84 and 14.20%, respectively (**Figure 5**).

## 4 Discussion

### 4.1 N uptake by rice plants in different stages

The present study showed that the total N uptake by rice plants of WRS and ORS was higher than that by plants of FRN, but N uptake decreased at 0–20 DAT (**Figure 1**). In paddy systems, rapid straw decomposition may also lead to a high accumulation of allelochemicals during early straw incorporation and cause rapid changes in soil properties, inhibit root growth, and decrease N uptake (Liu et al., 2007; Yan et al., 2019). Except for the allelochemicals, the decrease in N uptake by rice plants in the early growth stages may also be attributed to the following: i) Some amount of the N released by the base-tillering fertilizer was absorbed by the straw. Chen et al. (2022) found that the N content of mulched straw increases after fertilization, thus reducing the ammonia volatilization loss of fertilizer N. The results of the mini-plot experiment with ^15^N-labeled urea showed that fertilizer N in the undecayed straw was 1.55–2.97 kg/ha at the mature stage of rice, which indicated that a large amount of fertilizer N was absorbed by the straw in the early stage, and part of fertilizer N was not released into the soil until rice maturity. ii) After returning to the field, straw decomposes rapidly in the early stage, which rapidly increases the soil C content, leading to an unbalanced soil C/N ratio and resulting in competition for N between microorganisms and rice plants (Zhang et al., 2010; Kuzyakov and Xu, 2013). However, no-tillage with straw mulching increases soil bacterial community diversity (Luo et al., 2020) and the activities of soil enzymes, including invertase, acid phosphatase, and urease (Iqbal et al., 2021); improves soil N retention capacity, and reduces N loss risk (Yang et al., 2022b), all of which lead to more efficient N recycling in cropping systems (Yang et al., 2023). Therefore, WRS and ORS promoted N uptake by rice plants in the later growth stages (from HS to MS) and significantly increased the total N accumulation in rice plants under no-tillage with straw mulching conditions. This reveals that the straw mulching of dryland crops has the potential to reduce N input and increase rice yield under paddy-upland rotation with no-tillage.

### 4.2 N sources of rice plants with straw mulching

The results of this study showed that total N accumulation in rice plants increased in the WRS and ORS, which was mainly attributed to an increase in the uptake of soil N (**Figure 3**). Soil is the main N source for crop uptake, and approximately 50–80% of the N absorbed by rice during its entire growth period is sourced from the soil (Zhu, 2008). Additional N sources promote the absorption of soil N by plants, which was defined as the “priming effect” or “added N interaction” by Hamid and Ahmad (1993). Under the paddy-upland rotation, N application during the rice season increases the absorption of soil N from previous crops by rice plants (Zhou et al., 2020). In this study, the soil N uptake increased to 97.89, 124.15, and 119.18 kg/ha for FRN, WRS, and ORS, respectively. Straw mulching promotes the establishment of soil microbial colonies and increases fungal and enzyme activities (Bolinder et al., 2020; Dhaliwal et al., 2020), which significantly improves the mineralization and release of soil N, increases the transformation of ammonium and nitrate, and promotes the absorption of soil N by rice plants (Quan et al., 2018). In addition, although the straw released 20.31–34.97 kg/ha N in the rice season, there was little absorption by rice plants (**Figure 5**). Li et al. (2009) reported that more than 50% of the N in crop straw is refractory organic matter, which can only be absorbed by plants after transformation by microorganisms. Therefore, N released by straw is mainly retained in the soil during the short period of crop growth. This could explain why straw mulching not only increases N uptake by plants but also improves soil N content (Liu et al., 2021). The transformation and utilization of straw N require further study to reveal the mechanism of long-term straw return to improve soil fertility and crop yield.

## 5 Conclusion

The release, absorption, and utilization of straw N by rice under no-tillage and straw mulching conditions were investigated in the present study. It was revealed that wheat and oilseed rape straw mulching increased the total N uptake of rice by 2.99–29.67%. The amount of straw N absorbed by rice plants was 1.06–1.20 kg/ha, which is only 0.62–0.66% of the total N uptake; approximately 65.47–72.37% was from the soil indicating that straw mulching promotes the utilization of soil N to a higher degree than that of inorganic N fertilizer by rice plants under no-tillage wheat/oilseed rape–rice cropping systems. In addition, straw mulching increased the N utilization efficiency of tillering, panicle, and total fertilizer by 2.84–25.30%; however, base fertilizer was dependent on mulching straw. The results obtained in this study provide a theoretical basis for the effective utilization of straw and rational N application practices in paddy-upland rotations in the future. However, many aspects of straw mulching, in particular, how the microbial transformation process of straw N mediates the utilization of straw N, require further investigations.

## Data availability statement

The original contributions presented in the study are included in the article/supplementary material. Further inquiries can be directed to the corresponding author.

## Author contributions

FY and JM designed the research, FY performed the experiments. FY and WZ analyzed the data and wrote the manuscript. YjS, CG, KX, NL, ZY, YW and QZ provided assistance with sampling and investigation. YyS provided assistance with meteorological data collection. JM and XW revised the manuscript. All authors contributed to the article and approved the submitted version.
